# Network Pharmacology-Based Systematic Analysis of Molecular Mechanisms of *Geranium wilfordii* Maxim for HSV-2 Infection

**DOI:** 10.1155/2021/1009551

**Published:** 2021-11-03

**Authors:** Hao Zhang, Ming-Huang Gao, Yang Chen, Tao Liu

**Affiliations:** Department of Integrated Traditional Chinese and Western Medicine, Nanjing University of Chinese Medicine, Nanjing 210000, China

## Abstract

**Background:**

Being a traditional Chinese medicine, *Geranium wilfordii* Maxim (GWM) is used for the treatment of various infectious diseases, and its main active ingredients are the polyphenolic substances such as polyphenols quercetin, corilagin, and geraniin. Previous studies have demonstrated the anti-HSV-1 viral activity of these three main ingredients. Through employing a network pharmacological method, the authors of the present research intend to probe the mechanism of GWM for the therapeutic treatment of HSV-2 infection.

**Methods:**

The bioactive substances and related targets of GWM were obtained from the TCMSP database. Gene expression discrepancy for HSV-2 infection was obtained from dataset GSE18527. Crossover genes between disease target genes and GWM target genes were gained via Circos package. Distinctively displayed genes (DDGs) during HSV-2 infection were uploaded to the Metascape database with GWM target genes for further analysis. The tissue-specific distribution of the genes was obtained by uploading the genes to the PaGenBase database. Ingredient-gene-pathway (IGP) networks were constructed using Cytoscape software. Molecular docking investigations were carried out utilizing AutoDock Vina software.

**Results:**

Nine actively involved components were retrieved from the TCMSP database. After taking the intersection among 153 drug target genes and 83 DDGs, 7 crossover genes were screened. Gene enrichment analysis showed that GWM treatment of HSV-2 infection mainly involves cytokine signaling in the immune system, response to virus, epithelial cell differentiation, and type II interferon signaling (IFNG). One hub, three core objectives, and two critical paths were filtered out from the built network. Geraniin showed strong binding activity with HSV-2 gD protein and STING protein in molecular docking.

**Conclusions:**

This network pharmacological study provides a fundamental molecular mechanistic exploration of GWM for the treatment of HSV-2 infection.

## 1. Introduction

Genital herpes is a common sexually transmitted infection (STI) caused by herpes simplex virus type 2 (HSV-2) and represents a major health problem globally [1].

HSV-2 frequently modulates the cytokine milieu of the microenvironment in favor of HIV-1 spread [2]. The available antiviral agents used in HSV-2 infections are those that are clinically approved for the general treatment of HSV-2 infections, such as acyclovir and famciclovir. Indeed, previous studies of HSV-2 infection indicated that the use of single nucleoside analogues is inadequate for effective control of virus replication, as the administered nucleoside analogues often exert significant selection pressure on the virus, leading to the rapid generation of escape mutants. While current therapies based on nucleoside analogues suppress viral replication and reduce progression of HSV-2 infection, treatment is lifelong and viral cure is extremely rare [3]. Therefore, to further optimize treatment, new effective drugs are highly warranted.

GWM is a traditional Chinese medicine, and it contains geraniin, quercetin, corilagin, and so on [4]. Previous studies have demonstrated the anti-HSV-1 viral activity of these three main ingredients [5–7]. One study revealed a promising role of geraniin as an antiviral agent against HSV-2 infection with no apparent toxicity [8]. However, the mechanisms by which GWM inhibits HSV-2 infection remain unclear.

**Figure 1:**
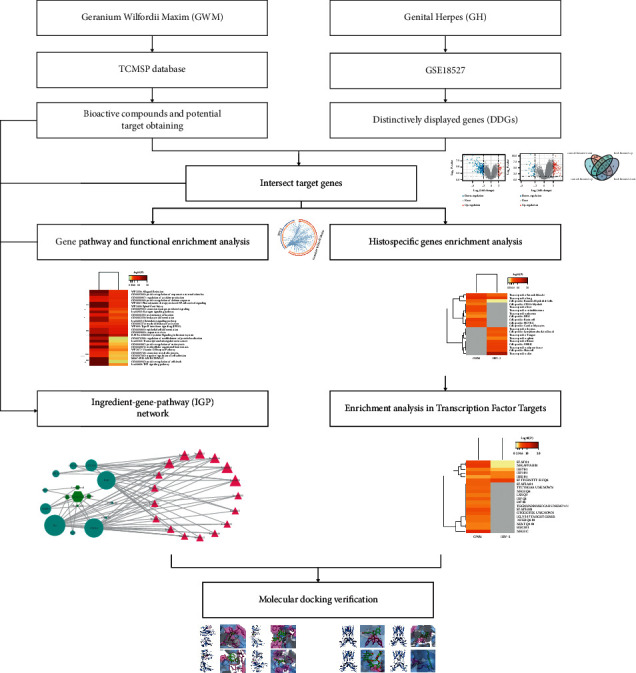
Workflow chart of *Geranium wilfordii* Maxim in the treatment of genital herpes based on network pharmacology.

Traditional Chinese medicine (TCM) has the characteristics of multitarget, multistep, and multilevel synergism [9]. Recently, network pharmacology becomes an important bioinformatics tool for identifying the mechanism of action of TCM [10]. In the present study, the network pharmacology approach was performed to further investigate the active ingredients and the underlying mechanism of GWM for the treatment of HSV-2. A flow diagram summarizing the different procedures of this study is shown in Figure 1.

## 2. Materials and Methods

### 2.1. Bioactive Chemical Substance and Objective Genes of GWM

Bioactive components and action targets of GWM were screened in the TCMSP website (old.tcmsp-e.com/tcmsp.php) [11]. Oral bioavailability (OB) ≥30% and drug-like properties (DL) ≥0.18 were the filtering criteria [12]. The active components were filtered using pharmacokinetic absorption, distribution, metabolism, and excretion guidelines (ADME) filter. Since the polyphenolic substance corilagin and geraniin are the main active ingredients of GWM, they are also listed. The active ingredient target genes were normalized in the UniProt database (uniprot.org). The structures of the active compounds were acquired at PubChem website (pubchem.ncbi.nlm.nih.gov).

### 2.2. DDGs in HSV-2 Infection

The GSE18527 dataset in the GEO database (ncbi.nlm.nih.gov/geo) was created by Peng T et al. The dataset had 19 samples, consisting of 3 cases of pretreatment healthy skin, 4 cases of pretreatment diseased skin, 6 cases of diseased skin in the healing group, and 6 cases of healthy skin in the healing group. The screening criteria for distinctively displayed genes were *P* < 0.05 and logFC >4. The DDGs were established by the intersection of the two datasets group: pretreatment healthy skin and pretreatment lesioned skin group and posttreatment diseased skin and pretreatment lesioned skin group.

### 2.3. Intersect Target Genes

Crossover genes between DDGs and GWM target genes were obtained using Circos software.

### 2.4. Gene Pathway and Functional Enrichment Analysis

DDGs during HSV-2 infection and GWM target genes were uploaded to the Metascape database (metascape.org) for further analysis of relevant genes and functional enrichment.

### 2.5. Histospecific Gene Enrichment Analysis

The distribution of genes was further analyzed after uploading DDGs during HSV-2 infection and GWM target genes to the PaGenBase database (bioinf.xmu.edu.cn/PaGenBase).

### 2.6. Enrichment Analysis of Transcription Factor Targets

To assess the potential regulatory patterns of the most enriched and conserved transcripts, the DDGs during HSV-2 infection and GWM target genes were submitted to transcription factor (TF) enrichment analysis by using the TRANSFAC Predicted Transcription Factor Targets dataset (https://maayanlab.cloud/Harmonizome/dataset/TRANSFAC + Predicted + Transcription + Factor + Targets). The obtained TFs were sorted according to their average enrichment scores. The top 20 TFs of both sets of mRNAs were further evaluated to determine their coregulatory network.

### 2.7. Ingredient-Gene-Pathway (IGP) Network

The IGP network was created by importing five intersecting active ingredients, seven intersecting genes, and the leading 17 KEGG pathways into Cytoscape software. The topological parameters such as degree centrality (DC), betweenness centrality (BC), and closeness centrality (CC) were utilized to evaluate the centrality features of nodes in IGP networks.

### 2.8. Molecular Docking Studies

The HSV-2 gD protein, STING protein, and drug structures were downloaded from the PDB (pdb.org) and PubChem websites. Drug and HSV-2 gD protein and STING protein docking investigations were conducted in AutoDock Vina (version 1.1.2) [13]. The visualization of the docking performance was performed by PyMOL v.2.3 software, and the docking effect was evaluated using the affinity value (AV).

## 3. Results

### 3.1. Bioactive Components and Drug Targets of GWM

Nine GWM bioactive chemicals were obtained in the TCMSP database, containing ellagic acid, sitosterol, kaempferol, furosin, ethyl brevifolincarboxylate, luteolin, quercetin, dehydrogeraniin, and corilagin. We downloaded the two-dimensional structure of the chemicals in PubChem website (Table 1). 309 drug targets were acquired in the TCMSP website and converted to target genes in the UniProt website. 153 target genes were isolated as drug-targeting genes after deleting repetitions.

### 3.2. DDGs in HSV-2 Infection

**Figure 2:**
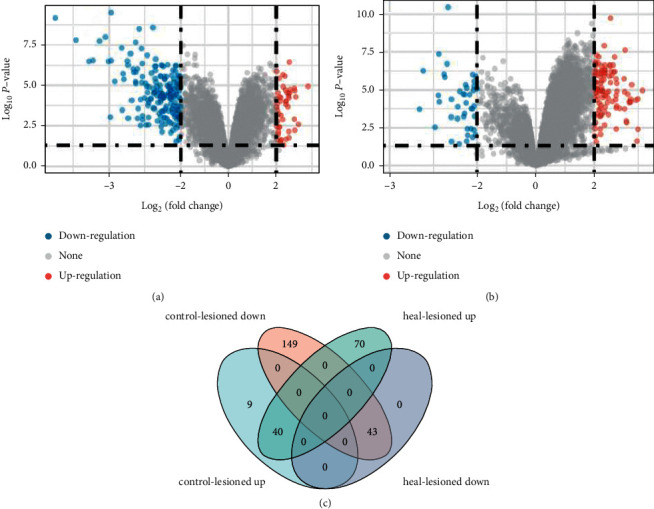
(a) Volcano map displays the differential genes between the pretreatment healthy skin and the pretreatment lesioned skin group. (b) Volcano map displays the differential genes between the posttreatment lesioned skin and the pretreatment lesioned skin group. (c) Venn diagram displays the intersection of the above 2 groups of differential genes. Up represents upregulated genes, and down represents downregulated genes.

Finally, 40 genes upregulated and 43 genes downregulated were selected. The Venn diagram and heat map of DDGs are shown in Figure 2.

### 3.3. Intersect Target Genes

**Figure 3:**
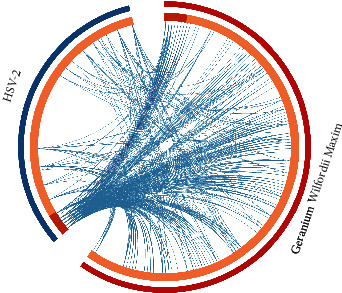
Red represents 153 gene targets of *Geranium wilfordii* Maxim; blue represents 83 genes significantly upregulated during HSV-2 infection; orange represents the intersection of the two groups of genes.

Seven crossover genes (CXCL10, CXCL11, CXCL8, IL-6 and IL-1*β*, MMP1, and SELE) were filtered in GWM target genes and HSV-2 DDGs via Circos package, as shown in Figure 3.

### 3.4. Gene Pathway and Function Enrichment Analysis

**Figure 4:**
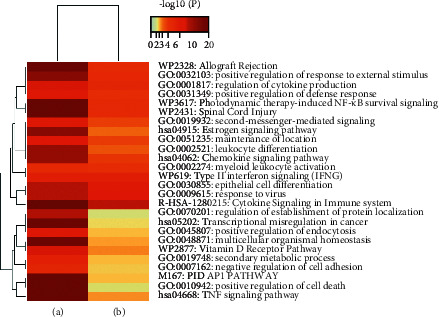
Gene pathway and functional enrichment analysis. (a) Enrichment analysis of 153 genes that are targets for the action of GWM. (b) Enrichment analysis of 83 genes that are significantly altered during HSV-2 infection.

The enrichment analysis of GWM target genes and DDGs during HSV-2 infection were jointly clustered in 26 enrichment items, and the most significant ones included cytokine signaling in the immune system, response to virus, epithelial cell differentiation, and type II interferon signaling (IFNG), as shown in Figure 4. These analyses suggested that GWM may treat HSV-2 infection by modulating cytokine signaling in the immune system, the process of cell state or activity changes due to viral stimulation, and the process by which unspecialized cells acquire specialized features of epithelial cells, the binding of IFNG to its receptor, and the subsequent phosphorylation cascade reaction involving the JAK and STAT protein families.

### 3.5. Histospecific Gene Enrichment Analysis

**Figure 5:**
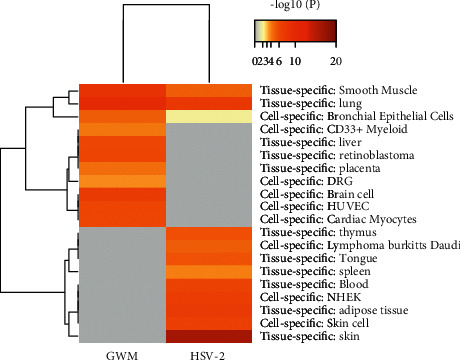
Summary of histospecific gene enrichment analysis in PaGenBase.

PaGenBase database tissue-specific enrichment profiling indicated that the target genes of GWM were mainly concentrated in lung and smooth muscle tissues; cell-specific was brain cell. The DDGs of HSV-2 were mainly enriched in skin tissue, followed by lung and smooth muscle tissues. Cell-specific was NHEK (normal human epidermal keratinocytes), as shown in Figure 5.

### 3.6. Enrichment Analysis in Transcription Factor Targets

**Figure 6:**
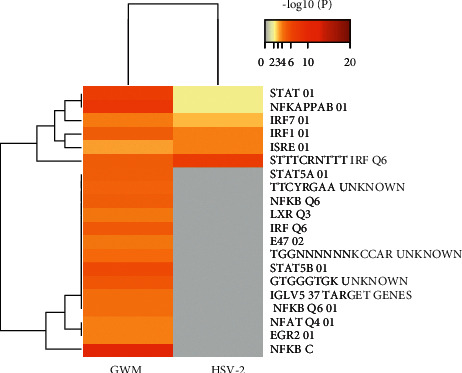
Summary of enrichment analysis in transcription factor targets.

Enrichment analysis of GWM target genes and HSV-2 DDGs transcription factors focused on STTTCRNTTT IRF Q6, ISRE 01, IRF1 01, IRF7 01, NFKAPPAB 01, and STAT 01, as shown in Figure 6. This suggested that GWM may regulate the ability of target genes during the progression of HSV-2 infection through changes in the activity or expression of the above transcription factors, thereby controlling HSV-2 infection.

### 3.7. Ingredient-Gene-Pathway (IGP) Network

**Figure 7:**
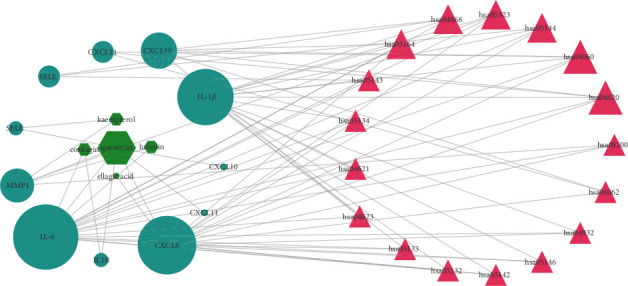
Ingredient-gene-pathway networks. Fuchsia triangles, aqua ellipses, and lime octagons stand for pathways of GWM, intersecting with target genes and active components, respectively. The bigger the shape of the graph, the larger the degree value of the node and the higher the role in the network.

The IGP network consists of 29 nodes (5 active ingredients, 7 intersecting genes, and 17 pathways) and 73 edges. In this network, we found that all 7 intersecting genes were related to quercetin, as shown in Figure 7. It is inferred that quercetin could be the pivotal effective component of GWM for the treatment of HSV-2 infection. According to the topological analysis, IL-6, IL-1*β*, and CXCL8 are the pivotal genes. Toll-like receptor signaling pathway and cytokine-cytokine receptor interaction pathway are the key pathways in the IGP network. Among them, IL-6, IL-1*β*, and CXCL10 are associated with the cytosolic DNA-sensing pathway. Since IFN*β* exerts a crucial function in the inhibition of HSV-2, HSV-2 has evolved multiple means to inhibit IFN*β* expression to produce immune escape [14–16], and the cGAS-STING pathway is a key mechanism for IFN*β* production [17]; it is hypothesized that GWM may act through IL-6, IL-1*β*, and CXCL10 in the cGAS-STING pathway as a key link in controlling HSV-2 infection.

### 3.8. Molecular Docking Studies

**Figure 8:**
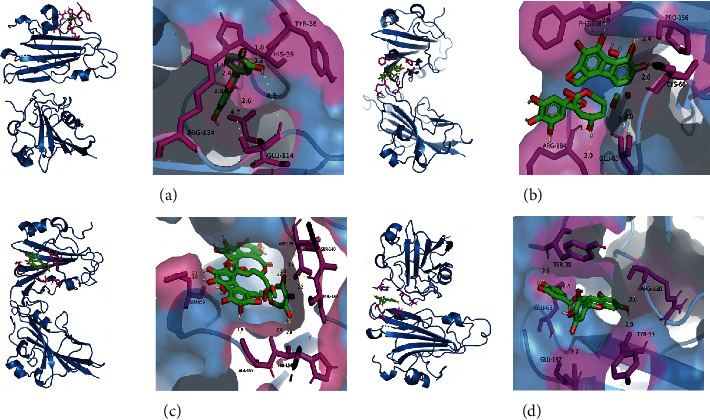
Molecular docking diagram. Molecular models of the binding of quercetin, corilagin, geraniin, and EGCG with HSV-2 gD protein, and the results are shown as 3D diagrams. (a) Quercetin-gD. (b) Corilagin-gD. (c) Geraniin-gD. (d) EGCG-gD.

**Figure 9:**
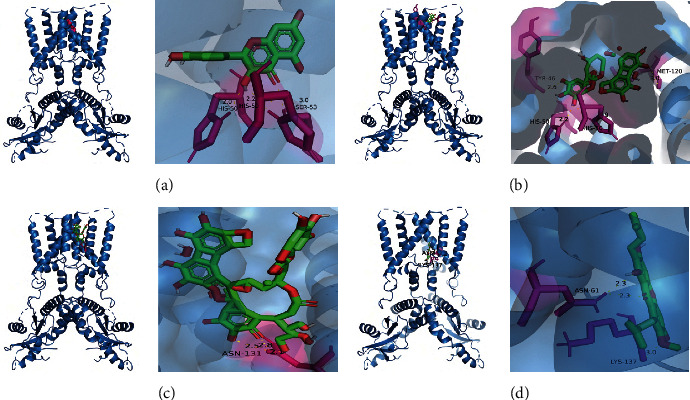
Molecular docking diagram. Molecular models of the binding of quercetin, corilagin, geraniin, and mangostin with STING protein, and the results are shown as 3D diagrams. (a) Quercetin-STING. (b) Corilagin-STING. (c) Geraniin-STING. (d) Mangostin-STING.

Nectin-1 is a cell adhesion protein, and binding of Nectin-1 protein by HSV-2 gD protein is necessary for HSV-2 to enter infected cells [18]. Epigallocatechin gallate (EGCG) has been shown to bind directly to HSV-2 gD protein to exert its anti-HSV-2 infection effect [19]. We docked quercetin, corilagin, geraniin, and EGCG to HSV-2 gD protein molecules respectively, and the binding activity of all the three was superior to that of EGCG, with geraniin showing the strongest binding activity (−17.44 kcal/mol), as shown in Table 2. cGAS acts as the primary intracellular double-stranded DNA (dsDNA) sensor, sensing intracellular dsDNA and generating the secondary messenger cGMP-AMP (cGAMP), which is further sensed by the sensing protein STING downstream of the interferon gene, leading to IFN*β* production [20]. HSV-2 has evolved multiple strategies to counteract this pathway, inhibiting IFN *β* production and evading host immunity [21]. Mangostin is a STING-targeted pathway agonist [22]. The binding activity of all the three was superior to that of mangostin, with geraniin showing the strongest binding activity (−11.71 kcal/mol), as shown in Table 2. These data suggested that quercetin, corilagin, and geraniin may affect the pathogenic process of HSV-2 by binding to the HSV-2 gD protein (Figure 8) interfering with the binding to the NECTIN receptor. The direct action of quercetin, corilagin, and geraniin with the cGAS-STING pathway relationship has yet to be experimentally verified. Molecular docking is shown in Figure 9.

## 4. Discussion

Five effective chemicals, namely, quercetin, corilagin, kaempferol, luteolin, and ellagic acid, were evaluated in the IGP network. Among them, quercetin exhibited the strongest node value, revealing that quercetin acts as a major element in the network. In a meta-analysis, quercetin-type flavonols were noted to have antiviral activity and significantly reduced the mortality of infected animals [23]. Three central genes were screened in the IGP network, including IL-6, IL-1*β*, and CXCL8, which were all linked to quercetin. We hypothesized that GWM may exert antiviral effects by regulating the above targets during HSV-2 infection.

IL-6 is produced by pathogen-associated molecular patterns (PAMPs) that stimulate cells such as endothelial cells, smooth muscle cells and immune cells to exert a wide range of tissue effects [24]. IL-6 can protect mice from HSV-2-induced mortality [25]. Estradiol-treated mice exhibited sooner recruitment and a larger ratio of Th1 and Th17 effector cells in the vagina and better protection after HSV-2 infection compared to placebo-treated controls, and Th17 responses were abolished in IL-1*β* knockout APC-T cells, suggesting that IL-1*β* is a crucial element in the induction of Th17 in the reproductive tract [26]. CXCL8, CXCL9, and CXCL10 were found to be expressed at high levels in both HSV-1 and HSV-2 CNS infections in one study [27].

We identified 2 crucial signaling pathways in the IGP network: toll-like receptor (TLR) signaling pathway and cytokine-cytokine receptor interaction pathway. TLR9 pathway specifically recognizes the unmethylated CpG motifs in dsDNA (CpG DNA) [28]. Studies have confirmed that using pattern recognition receptor (PRR) antagonists, such as lipoproteins, CpG DNA, and cyclic dinucleotides, we can greatly limit HSV-2 replication. TLR9 silencing also affects IL-6 secretion when HSV-2 or viral DNA stimulates the cells [29]. The cytokine-cytokine receptor interaction pathway, according to the annotation of the KEGG (genome.jp/kegg) database, is mainly the interaction between HSV-2 glycoprotein and chemokines such as (CCL26, CCL28, CCL22, CCL25, CXCL9, CXCL10, CXCL11, and CXCL13).

Therefore, we found that these signaling pathways are closely associated with HSV-2 infection. GWM may contribute to the therapeutic role of HSV-2 infection by modulating these signaling pathways. The constituents of GWM are highly sophisticated, and since it is impossible to include all of them in the database, some constituents and their targets of action could be overlooked.

## 5. Conclusions

The “multicomponent, multitarget, and multipathway” nature of GWM for HSV-2 infection was demonstrated in this study. Using a network pharmacology approach, we identified that quercetin acts on the targets IL-6, IL-1*β*, and CXCL10 through a key signaling pathway (toll-like receptor signaling pathway and cytokine-cytokine receptor interaction) as a key component in controlling HSV-2 infection. This work offers ideas for future research on the molecular mechanisms of GWM for the treatment of HSV-2 infection, and related studies can be seen in future research.

## Figures and Tables

**Table 1:** Active ingredients and ADME parameters of *Geranium wilfordii* Maxim (GWM).

NO.	Molecule ID	Molecule name	Chemical formula	Structure	MW	OB (%)	DL
1	MOL001002	Ellagic acid	C_14_H_6_O_8_	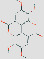	302.2	43.06	0.43
2	MOL000359	Sitosterol	C_29_H_50_O	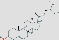	414.79	36.91	0.75
3	MOL000422	Kaempferol	C_15_H_10_O_6_	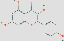	286.25	41.88	0.24
4	MOL005067	Furosin	C_27_H_22_O_19_	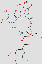	650.49	40.53	0.29
5	MOL005073	Ethyl brevifolin carboxylate	C_15_H_12_O_8_	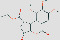	320.27	30.86	0.33
6	MOL000006	Luteolin	C_15_H_10_O_6_	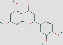	286.25	36.16	0.25
7	MOL000098	Quercetin	C_15_H_10_O_7_	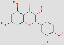	302.25	46.43	0.28
8	MOL005064	Dehydrogeraniin	C_41_H_28_O_28_	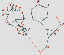	968.68	59.57	0.01
9	MOL005079	Corilagin	C_27_H_22_O_18_	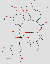	634.49	3.01	0.44

ADME: absorption, distribution, metabolism, and excretion.

**Table 2:** Docking scores of active ingredients of GWM with potential targets.

Targets	PDB ID	Compound	Affinity (kcal/mol)
HSV-2 gD	4MYV	Quercetin	−7.92
HSV-2 gD	4MYV	Corilagin	−13.08
HSV-2 gD	4MYV	Geraniin	−17.44
HSV-2 gD	4MYV	EGCG	−6.88
STING	6NT5	Quercetin	−7.1
STING	6NT5	Corilagin	−11.65
STING	6NT5	Geraniin	−11.71
STING	6NT5	Mangostin	−4.52

## Data Availability

All data are available from the corresponding author upon reasonable request.
